# Dietary recommendations for people with diabetes in special situations: a position statement report by Arabic Association for the Study of Diabetes and metabolism (AASD)

**DOI:** 10.1186/s41043-024-00619-y

**Published:** 2024-09-03

**Authors:** Amin Roshdy Soliman, Mona Hegazy, Rabab Mahmoud Ahmed, Shereen Abdelghaffar, Mohammed Gomaa, Sahar Alwakil, Dina Soliman, Lobna Sedky, Inass Shaltout

**Affiliations:** 1 Internal Medicine and Nephrology, Kasr Alainy Faculty of Medicine- Cairo University, Cairo, Egypt; 2 Internal Medicine, Hepatology, and Gastroenterology Division, Kasr Alainy Faculty of Medicine- Cairo University, Cairo, Egypt; 3 Pediatric Diabetes and Endocrinology, Kasr Alainy Faculty of Medicine- Cairo University, Cairo, Egypt; 4 Internal Medicine, Kasr Alainy Faculty of Medicine- Cairo University, Cairo, Egypt; 5 Internal medicine, Diabetes , Endocrinology and Clinical nutrition, Kasr Alainy Faculty of Medicine- Cairo University, Cairo, Egypt; 6 Department of Anesthesiology, Kasr Alainy Faculty of Medicine- Cairo University, Cairo, Egypt; 7 Clinical Oncology, Kasr Alainy Faculty of medicine, NEMROCK (kasr Al Ainy Center of Clinical Oncology), Kasr Alainy Faculty of Medicine, Cairo University, Cairo, Egypt; 8 Woman4Oncology-Egypt organization: W40-E. Co-supervisor of Nemrock Onco-Nutrition MDT, Cairo, Egypt; 9 Internal Medicine and Diabetes, Kasr Alainy Faculty of Medicine, Cairo University, Cairo, Egypt

**Keywords:** Diabetes, Diet, Egypt, Arab countries

## Abstract

**Background:**

Diabetes is a significant global health concern. Regional factors play a crucial role in determining the appropriate diet for patients.

**Main body:**

The Arabic Association for the Study of Diabetes and Metabolism has developed a position statement that addresses the dietary needs of patients in the context of low income and cultural dietary habits. This statement aims to explore the most suitable diet for Middle East and North Africa (MENA) region and provide guidance for physicians to overcome barriers in optimal care. While most dietary guidelines focus on uncomplicated diabetes, it’s essential to recognize that diabetes often coexists with other common diseases in our region.

**Conclusion:**

International guidelines cannot be directly applied to the Egypt and Arab countries due to cultural and dietary differences. Our position statement shares valuable insights into managing diabetes in special situations and diverse clinical settings within this region. These recommendations are flexible, considering personal, cultural, and traditional differences.

## Introduction

Diabetes is a significant global health concern. Currently, about 537 million adults aged 20–79 years live with diabetes worldwide, accounting for 10.5% of this age group’s population. By 2030, the number of people with diabetes is expected to increase to 643 million (11.3%), and by 2045, it may reach 783 million (12.2%) [1]. In the Middle East and North Africa (MENA) region, diabetes mellitus (DM) has the highest regional prevalence, ranging from 16.2 to 24.5%. It is also expected to experience an 86% increase, reaching a predicted 136 million cases by 2045 [2]. In Egypt, the total number of adults with diabetes in Egypt in 2021 between 10 and 20 million with 6.7 million adults are estimated to have died due to diabetes or its complications in 2021 [3]. Despite being home to 13.6% of people with diabetes worldwide, only a little was spent on diabetes in this region [2].

Given that type 2 diabetes is primarily considered a diet-related chronic disease and considering the challenges in the Egypt and Arab countries related to healthcare accessibility and quality (which are less efficient compared to European and Western Pacific regions), and in the presence of the documented scientific connection between food and health, it becomes essential to prioritize dietary interventions as a simple low cost means of treatment.

Most of Dietary Guidelines primarily focused on patients with uncomplicated diabetes. However, it’s important to recognize that diabetes often coexists with other common disease in our region diseases. For instance, it can either cause certain conditions (such as kidney disease) or occur alongside them in the same patient like liver diseases, cancer which may coincide with diabetes. International guidelines cannot be simply, directly, and blindly applied everywhere. Moreover, access to clinical nutritionists who are experts in diabetes as well as to diabetes educators with medical background is not always widely available in this region, different from the case in Europe and United States of America [3].

## Methods

### Objectives

The primary objective of this position statement is to enhance awareness among individuals with diabetes and healthcare professionals about nutrition strategies that can be advantageous in specific diabetes-related scenarios. The scope includes recommendations tailored and adapted to suit dietary habits and preferences taking into consideration the socioeconomic status. Achieving this goal involves utilizing the most reliable scientific evidence while considering treatment objectives, strategies for achieving those objectives, and the adjustments that individuals with diabetes are willing and able to implement.

This paper provided our own experience regarding diet in diabetic individuals in special situations and in different clinical settings in Egypt and Arab countries from An Arabic Association for the Study of Diabetes and metabolism point of view to provide a practical recommendations and tips in one place for general health care practitioners who treat people with diabetes.

These recommendations are adaptable to address the unique requirements of individuals with diabetes. They offer a customizable structure comprising essential components that allow individuals to tailor their approach, considering personal, cultural, and traditional variations, as well as the specific challenges faced in our region.

### Expert Panel

Experts were selected based on their extensive experience in their sub-specialties and their contributions to relevant research and clinical practice.

The panel comprised 9 experts, including endocrine, hepatology, nephrology, geriatric, critical care, and clinical oncology. Panel members were responsible for searching, reviewing the evidence, contributing to the summarization to create an overview of the available recommendations, and reaching consensus. The Delphi method was utilized to achieve consensus, comprising iterative rounds of discussion, and voting on critical recommendations. All panel participants disclosed any potential conflicts of interest, which were thoroughly reviewed and appropriately managed to ensure impartial and unbiased recommendations.

### Evidence review

This position statement utilized a focused comprehensive approach by searching existing databases, utilizing keywords including, but not restricted to (diabetes, diet, chronic liver disease, alcoholic liver disease, non-alcoholic fatty liver disease, non-alcoholic steatohepatitis, autoimmune hepatitis, chronic kidney disease, diabetic kidney disease, end stage renal disease, dialysis, elderly, geriatric, children, adolescents, pregnancy, cancer, malignant, intensive care and critical care) from searches developed by group of specialists. The researchers conducted a systematic search across databases, thoroughly reviewing all relevant citations From Egypt and Arab countries, with a particular focus on full-text publications from the past decade. Additionally, they identified further relevant citations by examining the references of the articles they had already identified. All the experts screened titles and abstracts, followed by a full-text review to determine final inclusion.

### Consensus process

The consensus process was crucial to ensure that the recommendations accurately represent the collective judgment of the expert panel. In the initial round, each expert provided input on draft recommendations based on their review of the evidence. Subsequent rounds involved aggregating feedback, discussing divergent views, and making necessary revisions to the recommendations. Experts voted on each recommendation using a Likert scale to indicate their level of agreement. Consensus was defined as at least 75% of experts agreeing on a recommendation. Disagreements were addressed through additional discussions and revisions until consensus was reached. All discussions, votes, and revisions were meticulously documented to ensure transparency and accountability.

### Drafting and Review

The initial version of the position statement was prepared by the lead authors, who synthesized evidence and preliminary recommendations from the expert panel. This draft was then shared with other expert panel members for detailed review and feedback. External reviewers, including additional experts in the field and stakeholders, were also invited to review the draft and provide input. Feedback from both internal and external reviews was systematically incorporated into subsequent drafts, with revisions made to address key comments and suggestions. Multiple rounds of review and revision were conducted to refine the recommendations and ensure clarity and accuracy. Given the limited availability of high-quality evidence in our region, consensus recommendations were formulated, and no formal grading or independent methods review process was conducted.

## Diet for people with diabetes in liver disease

Liver diseases have been a substantial public health concern in the region, impacting millions of people over the years. The distribution of the various etiologies for these diseases has been gradually changing, with the proportion of virus-induced hepatitis infections declining, while the proportion of non-alcoholic fatty liver disease (NAFLD) increasing [4].

Patients with chronic liver disease are generally advised to consume a healthy, varied diet, avoiding processed food, industrialized foods, sugar-sweetened beverages, and high-fat foods [5].

We had previously investigated the role of conjugated linoleic acid (CLA), influences of energy metabolism regulation. CLA is naturally present in dairy products (milk, yogurt, and cheese), eggs, veal, and lamb from ruminant grass-fed animals. Our previous research has shown a marked deficiency of CLA in patients with DM and MASLD. Therefore, our recommendation is to consume foods naturally enriched with CLA, not as supplements, due to the potential for harmful effects. So, we recommend incorporating the above listed food into the typical Egyptian diet to modulate CLA levels [6].

Eating Habits and Disease Outcomes: According to our previously published data during the COVID-19 crisis, the eating habits of the Egyptian population had an impact on disease outcomes [7, 8]. For the Healthy Eating Index (HEI-2015), which measures 13 constituents reflecting the recommendations in the 2015–2020 Dietary Guidelines for Americans, including two groupings: Adequacy components (“nine food types we should eat enough in sufficient quantities”) [9], none of the Egyptians exceeded a score of 71 out of 100, and the majority were between 35 and 56 out of 100.

Furthermore, none of the Egyptians consumed the recommended daily fiber intake of 25 g for women and 38 g for men, indicating a poor prebiotic intake. American Dietary Guidelines in 2010 stated that foods high in prebiotics, such as beans, wheat, onions, artichoke, barley, banana, tomato, and soybeans, should be consumed to meet the recommended daily fibers amount [10]. It is worth noting that fava beans, a traditional breakfast in many countries in our region, are consumed by all socio-economic classes and contain 9.03 g of fiber per 100 g [11].

Probiotic foods are based on the number of live organisms; thus, 100 g serving yogurts contain > 106 colony-forming units (CFU) per ml, will provide sufficient probiotic bacteria. However, only one product provided this among all the commercial yogurt brands in the Egyptian market, and for sorry information on the type of bacteria (Bifidobacterium), and CFU values were not provided on the packaging of either the full-cream or skimmed type. Bassuoni et al. have determined that a single 135 g daily portion of probiotic yogurt (PY) provided 1.4 × 109 CFU of Bifidum bacteria [12].

Screening and Management of NAFLD: European guidelines recommend that NAFLD patients should be screened for diabetes, and patients with type 2 DM must be screened for the presence of NAFLD, irrespective of serum transaminases.

Diabetes risk and overt type 2 diabetes are associated with more severe NAFLD, progression to non-alcoholic steatohepatitis (NASH), advanced fibrosis, and the development of hepatocellular carcinoma [13]. Lifestyle and profound improvement of steatosis, inflammation, and ballooning were observed when weight loss of > 7–9% was achieved, while only a weight loss > 10% was associated with improvement in fibrosis. A Mediterranean diet (MedD) should be advised to improve steatosis and insulin sensitivity [14].

## AASD position statement

Dietary Recommendations for Metabolic Dysfunction-Associated Steatotic Liver Disease (MASLD) Patients with Diabetes in the Egypt and Arab countries:

1. For MASLD patients with diabetes in the MENA region, the individualization of macronutrient composition will depend on the metabolic status of the individual (including lipid profile and renal function).
2. Carbohydrates should contribute 40–45%, with fat contributing 35–40%, mainly from polyunsaturated and monounsaturated sources (PUFA and MUFA). MUFA has been shown to improve glycemic control and cardiovascular disease risk factors in individuals with type 2 diabetes, with an equal percentage of omega-3 and omega-6 fatty acids.
3. Seafood (fish) should be introduced into the diet at least three times weekly to ensure the Mediterranean diet recommendation, and an animal source of omega-3 (covering EPA, DHA, and linoleic acid) is recommended as anti-inflammatory micronutrients for hepatic steatohepatitis (MASH).
4. One serving of saturated fat from an animal source, such as 200 ml of full-cream milk or yogurt, should be included to obtain the recommended conjugated linoleic acid (CLA).
5. The recommended daily fiber intake must be achieved by advising the consumption of more raw vegetables and fruits within the allowed percentage of carbohydrates (five different types daily), with the addition of protein and fiber from fava beans, a beloved traditional food, and whole-grain bread. One carbohydrate exchange (or serving) is a portion of food containing 15 g of carbohydrate.
6. Nutrition Education and Awareness: Simple nutrition education campaigns are needed to inform the public about the benefits of a Mediterranean-style diet, the dangers of trans fats, processed and ultra-processed foods, and the importance of minimizing fast food and sugary beverages. Awareness of the glycemic index and load of different food types is also crucial. Dietary Recommendations for Patients with Diabetes and End-Stage Liver Disease in the MENA Region:
7. For patients with diabetes and end-stage liver disease in Egypt and Arab countries, periods of starvation should be kept short, with a maximum of 4 h, due to the defective breakdown of liver glycogen and release of glucose for energy. Consuming 5 meals per day and a late evening snack should be recommended to improve total body protein status.
8. Non-malnourished patients with compensated cirrhosis should ingest 1.2 g/kg/day of protein, while for malnourished and/or sarcopenic cirrhotic patients, the amount should be increased to 1.5 g/kg/day of protein. Animal-source high-biological-value proteins, containing all the essential amino acids that the body cannot synthesize, should be encouraged in the diet (e.g., milk, eggs, meat, and poultry) to cover the needs of beneficial branched-chain amino acids (BCAAs).
9. Peanuts, a traditional nut in Egypt that is cheap and contains the needed BCAAs, should be recommended daily in a non-salted form, especially for patients with ascites.
10. Patients who are intolerant to animal protein or during the first 48 h of hepatic encephalopathy should consume a combination of two types of vegetable proteins (low biological value proteins), such as fava beans, lentil soup, chickpeas, soybeans, and kidney beans, daily.
11. Sweet potato, a starchy vegetable high in complex carbohydrates, is a perfect source of energy and glucose and can be recommended as a midnight snack.
12. With low-sodium diet, the increased risk of lower food consumption due to unpalatability should be balanced against the moderate advantage in the treatment of ascites.

### Diet for people with diabetes in kidney disease

Chronic kidney disease (CKD) and diabetes mellitus (DM) are closely related conditions; diabetes is the main cause of end-stage renal disease (ESRD) and accounts for about 22% of CKD patients [15]. The prevalence (ESRD) in MENA and Gulf Cooperation Council (GCC), ranged between 55 and 818 per million populations, 30% up to 42.5% of which is attributed to diabetic kidney disease (DKD) [16, 17]. In countries with limited resources, DKD has a financial burden. To limit treatment cost, primary and secondary prevention of chronic renal disease are essential [16].

Whether a healthier diet pattern will affect albuminuria, DKD progression is unclear, strong evidence are there to prove that, the current Western dietary pattern, enriched in animal protein, total and saturated fat, salt and simple sugar had been strongly linked to many chronic diseases exacerbation including DKD [18]. However, proper diet proved to slow chronic renal disease progression and will prevent its complications, but the steps taken to address these concerns are considerably minimal in our region [19].

There are, undoubtedly, regional factors that may affect the proper diet which should be adapted to both the patient and population habits to improve adherence rate which in turn will affect the patients’ outcome [20]. Dietary modification in this group of patients is challenging as people with DM are exposed to more protein in expense of carbohydrate and lipids to control hyperglycemia. This will be faced by electrolyte, acid-base imbalance and to the kidney’s ability to deal with this protein and its associated phosphorus load which varies again according to DKD stage [19].

A little is known about the exact quantity and the quality of dietary protein among DKD patients and no clear recommendations for that even in the most updated guidelines. Nutritional studies including Randomized controlled studies, Cochrane review and meta-analyses examining protein restriction have produced conflicting results due to a differential effect of the type of diabetes, early Vs late DKD stages, variation in outcome measurements for kidney function, missing of diet adherence testing [21–23]. No long-term studies to prove the validity of any assumption. Low dietary protein intake of 0.8 g/kg of body weight/day is recommend by most expert panels [19, 24]. In our region, real world studies revealed that, most of diabetic CKD patients not on dialysis consume variable amounts of dietary protein but unfortunately, published studies are deficient in giving data about the source or composition of different protein types consumed by those patients [20, 25].

Since both diabetes mellitus and chronic kidney disease are common disorders, patients with both conditions may or may not have DKD. No published research are found studied the proper protein intake in nephrotic syndrome associated with DM [24] and even general studies found discussing this issue in nephrotic syndrome due to any cause fail to define the minimum safe protein intake for the degree of proteinuria or in the presence of catabolic state [26].

Studies on Plant based protein in DKD patients are sparse but so far it may be beneficial for them taking into consideration the associated potassium derangements and, vitamin B and D deficiency [27, 28].

Considering the diverse nature of obesity, which varies in terms of severity, the presence of sarcopenia, and comorbidities; the question of adjusted versus real body weight for calculation of protein intake is crucial. Obesity is prevalent in diabetes and in CKD patients [29], low carbohydrate (LC) diets < 20 gm/d in patients with underlying diabetic kidney disease was safe in some studies but evidence of benefits or risks of LC diets is not strong enough [30]. However, definitive recommendations for dietary carbohydrate and fat are not yet available for DKD patients. In general whole-grain carbohydrates, fiber, and fresh fruits and vegetables are recommended for them. However, the quantity of portions and the specific food choices from these groups often require adjustments based on their potassium content, an attention to the way of food preparation to minimize hyperkalemia by re-boiling, re-cooking the vegetables is crucial. Growing evidence suggesting beneficial effects of omega-3 fatty acids on albuminuria in diabetic nephropathy [31], avocados, nuts, and olive oil, can help in regards this issue.

The primary source of salt in Egypt comes from bread, pickles, some types of salty smoked (Renga) or pickled fish (Feseekh) and salty cheese (Estanbouly or Mish) not from adding table salt to cooked food. Current nutritional guidelines for DKD patients recommend restriction of dietary sodium intake to less than 2 g/day (5 g of sodium chloride) [19]. However, some studies have reported that excessively low sodium intake adversely affects various physiological processes such as glucose metabolism, insulin sensitivity and renin–angiotensin–aldosterone system and has been associated with increased mortality [32].

In pre-ESRD DKD patients, a meta-analysis include small, heterogeneous studies found that ketoanalogue supplementation with a very low protein diet may ameliorate eGFR decline, reduce proteinuria, reduce serum urea levels and reduce the mortality [33] .No published studies were found discussing the clinical outcomes for using ketoanalogues or amino acids either with very low protein diet or with plant based diet in our region and unfortunately, most of patients receive wrong low dose of ketoanalouges and the reason for this is the very high price of these drugs in the market.

No studies are published in our region discussing intra-dialytic feeding in DKD patients. Whether these patients are permitted to eat while receiving dialysis or not remains a rather controversial issue due to high prevalence of intradialytic hypoglycemia and symptomatic hypotension in diabetic patients [34].

### **AASD position statement**

1. Although there are religious and cultural similarities among Egypt and Arab countries, substantial economic disparities persist. When prescribing diets for individuals with diabetes mellitus (DM) and kidney disease, it is essential to tailor them to each patient’s specific circumstances. Factors such as cost, taste preferences, comorbid medical conditions, and religious and cultural dietary practices should be considered.
2. Among pre-dialysis DKD patients, we recommend avoidance of high protein intake, and we suggest protein intake as the same general population with eGFR > 60 ml/min with a modest protein restriction with a target daily protein intake 0.6–0.8 g/kg /d with eGFR < 60. Protein restriction should be weighed against malnutrition and in cases with diabetic foot, ulcers and wounds, higher amount can be allowed for better healing. Very low-protein intake is not recommended (< 0.4 g/kg/day) due to the associated higher mortality, high prevalence of malnutrition and high non-compliance rate to the proper dose of ketoanalouges. We do not restrict protein intake among patients with DM and nephrotic syndrome with normal kidney function. We recommend use of adjusted body weight for protein intake calculation.
3. Plant based diets may be suitable for patients in our region because it matches our eating style and it have less cost than animal-based sources provided consumption of different types of plant-based proteins and high biologic protein should be taken in moderation preferred lean protein (eggs and chicken are good sources) with less milk, dairy products, processed food and fish.
4. sodium restriction should be individualized, according to patients’ blood pressure, volume status, proteinuria to lower blood pressure (BP), the rate of progression CKD stage.
5. We recommend use of a very low-protein diet supplemented with ketoanalogues of amino acids for conservative treatment only if they used in a proper dose and when indicated as in elderly patients, risk of malnutrition, good compliance, low serum albumin, and with vegan diets otherwise the patients can follow low protein diet (LPD).
6. Intra-dialytic feeding can be allowed in people with DM and ESRD if there is a clear net clinical benefit with avoidance of cola (high levels of phosphorus or juices that contain high levels of potassium. If hypoglycemia occurs in advanced stages of CKD or during dialysis, they can use honey or low potassium juice or fruit such as apple.

## Diet for children and adolescents living with diabetes

Although all evidence-based guidelines highlight the importance of nutritional requirements in young people with diabetes, many issues in dietary management for youth with diabetes are based on expert opinion. Additionally, some aspects remain debatable. Nutrition interventions and meal plans should be individualized and customized to accommodate cultural and traditional preferences [35, 36].

There are a lot of debatable items regarding nutritional management in children and adolescents with Diabetes. Using low carbohydrate diets in T1D has been recently and commonly adopted by many families [37]. Currently, there is lack of scientific evidence for use of very low carbohydrate diets in children and adolescents with T1D. Moreover, carbohydrate restriction can lead to dyslipidemia, ketosis, as well as disturbance of blood glucose levels. It can also restrict intake of essential nutrients, energy, and fiber which result in growth failure and delayed puberty [38].

Calculating carbohydrate, either by carbohydrate counting or choice, is important for optimizing glycemic control [37–39]. The use of glycemic index and glycemic load provides additional benefit for glycemic control over that observed when total carbohydrate is considered alone [38–40]. In people receiving basal bolus insulin therapy, adjusting insulin dose to amount of carbohydrate intake leads to better metabolic control and quality of life, with greater flexibility [41]. Consistency in carbohydrate amount is mandatory for those who apply fixed insulin doses to decrease glucose variability [42]. There are several methods of carbohydrate counting (15 g carbohydrate exchanges, 10–12 g carbohydrate portions, weighing food and multiplying by carb factor); with lack of evidence supporting that one method is superior to another. Insulin-to- carbohydrate ratios can be calculated using the 450–500 rules [43].

Although fat and protein have an influence on postprandial glucose, there is no agreement on the best way for their counting to match insulin doses [44]. Meals high in either protein or fat increase blood glucose up to 3–6 h after the meal and may lead to hypoglycemia 1–2 h. after eating. A formula based on fat protein units (FPU) and the Food Insulin Index (FII) has been proposed for protein and fat counting [45, 46]. Continuous glucose monitoring (CGM) is a useful tool for demonstrating the impact of certain foods and specific meals on glucose levels to clinicians as well as to patients [47].

A third debate is regarding the use non -nutritive sweeteners instead of sucrose to minimize insulin doses and decrease hyperglycemia and complications [48]. However, this is discouraged as it discriminates the child, and the long-term safety is unknown. Sucrose and sucrose-containing food and fluids should be consumed in limited amounts [49]. Sucrose can be substituted for other carbohydrate sources in the meal plan or, if added, covered with insulin. Sucrose can provide up to 10% of total daily energy intake [50, 51]. Sucrose sweetened beverage consumption has been associated with excessive weight gain [52]. Sucrose may be used instead of glucose to prevent or treat hypoglycemia [51]. Moreover, artificial sweeteners can be more expensive due to the cost of the ingredients, may be high in fat and may contain sweeteners with laxative effects such as polyols (sugar alcohols). There is lack of specific recommendation on the percentage of sugar or mono- or disaccharides in the diet in many countries including those of Egypt and Arab countries [50].

Nutritional advice on how to successfully manage both regular and unanticipated physical activity; and how to meet individual goals in competitive sports is recommended. Main recommendations are [51]: Pre-activity meals (1–2 h before exercise) should be relatively high in carbohydrate, moderate to high in protein, and low in fat. During exercise lasting more than 30 min, 15–30 g of carbohydrate should be consumed every 30–60 min [52]. Maintaining hydration by drinking around 150 ml of fluid every 15 min is advised [49]. A high-quality protein meal should be consumed after training [50].

### Considerations for special age groups

- Toddlers: Frequent, small meals over the day are more applicable due to inconsistent appetite; this can provide the nutritional requirements with better metabolic control. It is advisable to administer insulin before meals, but if not possible, splitting of the dose before and during feeding can be helpful [49].
- School aged Children: Managing diabetes in school setting requires a high degree of cooperation between all school staff, whether medical or non-medical, with children living with T1D and their families. Continuous Diabetes education and training is mandatory for all school personnel to apply safe and effective management strategies [48]. Moreover, healthy food choices should be available at school cafeterias [47].
- Adolescents: Adolescents resist any supervision on food choices and like to enjoy freedom and independence on content and timing of meals, which can negatively affect their metabolic control and general health [52]. The importance of receiving nutritive meals and avoiding frequent afternoon or evening snacking should be emphasized, to meet the demands of rapid growth and development. Insulin and meal timing should be individualized and customized according to variable school, exercise, and work schedules [53].

### AASD position statement

1. Individualized meal plans for youth with diabetes, provided by nutrition specialists, and supported by schools and media, should include a wide variety of healthy food choices, to meet the nutritional demands and the recommended daily allowances of different nutrients.
2. Low Carbohydrate diets should be discouraged to use in children and adolescents as it has many complications on growth, development, and overall health.
3. Fats and protein counting formulas studied in adults should be tried and validated in children.

### Diet in elderly with diabetes

Diabetes prevalence among the elderly people is increasing as the world is aging in all countries and despite the rate of population aging differs from country to another, a double fold increase in the ratio between elderly adults exceeding 65 years and the younger ages is expected in the upcoming 40 years in many countries [54]. According to the IDF the prevalence of diabetes mellitus in the elderly in Middle East and North Africa is about 24.2% [55].

Diabetes is strongly linked to frailty, which increases the risk of physical impairment related to falls and fractures increasing hospital admissions, and death rates due to decline in the reserve of organ function with aging. Diabetes can also lead to cognitive impairment and dementia which with frailty can strongly predict mortality in older people with diabetes, so it must be considered in the care of older adults with diabetes [56]. A meta-analysis revealed that in the Middle East prevalence of pre-frail reaches up to 39% while frail is 35% [57]. A systematic review on cognitive decline Epidemiology in Arabic region showed that Diabetes mellitus comes on top of the list of factors causing rapid progression of cognitive decline in Arabic region [58].

Contrary to expectations, studies showed that obese patients have a lower risk of cardiovascular events or death in comparison with normal weight patients, and this is known as “obesity paradox.” [59] However, some studies in the Arab region have revealed that BMI is a weak risk factor for cardiovascular comorbidities [60, 61]. In the Middle East, Metabolic syndrome is considered a major risk factor for worsening of atherosclerosis and cardiovascular disease in normoglycemic people and those with diabetes, but this association was rarely studied among elderly people with diabetes [62].

Sarcopenic obesity, defined by the coexistence of sarcopenia and obesity [63], is prevalent in people with diabetes. The increased risk of mortality due to cardiovascular diseases and atherosclerosis is strongly related to insulin resistance and inflammation, both can be accelerated in sarcopenic obesity with fat deposition and loss of muscle mass. Sarcopenic obesity has also been linked to cognitive impairment [63]. So, malnutrition is a red flag in elderly people with diabetes and should be managed carefully as the prevalence of malnutrition in elderly people with diabetes in some countries in our region may exceed 40% [64, 65].

Vitamin D deficiency is common in the Middle East and North Africa region [66] in the elderly and may affect various health outcomes, however vitamin D supplements’ effect on controlling type 2 diabetes is debatable [67, 68]. Proper nutrient intake in old age requires good oral health, as tooth loss can lead to poor consumption of essential nutrients [69].

Guidelines recommend consuming 2 or more servings of oily fish per week to obtain sufficient omega-3 polyunsaturated fatty acids, which may prevent cardiovascular events. A meta-analysis found that ω-3 FA are associated with improved inflammatory biomarkers and lipid profiles in diabetic and cardiovascular patients [70]. However, conflicting results from large-scale trials exist regarding cardiovascular benefits. Additionally, the safety of omega-3 supplements remains debatable, as they may increase the risk of atrial fibrillation in people with diabetes [71].

### AASD position statement

1. Elderly people with diabetes require around 30 kcal /kg /day, with individual adjustments based on nutritional status, physical activity, and other conditions.
2. Protein requirements up to 1.2–1.5 g /kg /day are essential to lower the risk of frailty and improve physical functioning.
3. Vitamin D supplementation if deficient, to improve bone health, muscle mass, and strength.
4. Consumption of unprocessed meat, dairy, eggs, fresh vegetables, and fruits to.
5. ensure adequate intake of antioxidant vitamins (C, E, carotenoids) and B- vitamins.
6. omega-3 rich foods as oily fish are required to adjust triglycerides, reduce inflammation, thus decreasing cardiovascular risk. Supplementation with EPA and DHA (2000–3000 mg/day) may benefit those with previous cardiovascular events.

### Diet for people with diabetes in pregnancy

Diabetes in pregnancy can be pre-gestational or gestational. Gestational diabetes (GDM) is defined as any degree of glucose intolerance starting with pregnancy. GDM managed without medications and only by dietary modification is called diet controlled GDM (A1GDM), while GDM managed with medications in addition to dietary modification is called type 2 GDM (A2GDM) [72].

Normal pregnancy is characterized by hormonal adaptations to ensure sufficient glucose availability for the growing fetus. Hyperglycemia is an important cause of various maternal and fetal complications, making glycemic control in diabetic mothers extremely important [73]. The prevalence of GDM varies widely, with reported rates ranging from 1 to 14% of pregnancies worldwide and 4.7–15.5% in the Middle East and North Africa, including 13.4% in Upper Egypt and 6–8% in North Egypt [74, 75].

Calorie allocation based on pre-pregnancy body weight, with about a 33% caloric reduction should be considered for weight gain control and glucose level management [76]. Approximately half of the calories should come from healthy carbohydrates, with a focus on low glycemic index foods to reduce the risk of.

macrosomia [76, 77].

Protein intake in pregnancy is important to prevent depletion of maternal stores and prevent muscle breakdown to accommodate the fetal demands [78].

Dash diet is a diet rich in fruits, vegetables, whole grains and low-fat dairy products and with small amounts of saturated fats and refined grains. Patients with GDM on dash diet had better metabolic outcomes and their infants have significantly lower birth weight. Mediterranean diet has proved effective to decrease the prevalence of GDM and decrease maternal and neonatal complications [79].

Vitamin D has a role in controlling insulin sensitivity. Trials have shown that Vitamin D supplementation decreases the risk of glucose intolerance and improves insulin resistance in those patients [80].

Inositol belonging to the group of B-complex vitamins. Myo-inositol supplementation in diabetic pregnant ladies is proved to decrease serum levels of glucose and insulin [81].

Exercise helps overcome insulin resistance and control fasting and postprandial hyperglycemia in gestational diabetes. Walking fast after main meals is often recommended [82].

Fish oil (omega-3) supplementation may have a beneficial impact on glucose and lipid metabolism, as well as inflammatory indices [83].

### AASD position statement

1. The total caloric recommendations are 30 kcal/kg in women with normal BMI, 24 kcal/kg in overweight women and 12–18 kcal/kg in obese mothers. Add another 500 kcal/day to the above recommendations in twin pregnancy.
2. The recommended daily energy intake of macro-nutrients is 50–55% complex carbohydrates, 25–30% fat and 12–20% proteins and 20–35 g/day fiber intake. At least 175 g/day of carbohydrates is essential for diabetic pregnant women.
3. Most nutritional guidelines recommend a protein intake between 10 and 20% of daily intake. Protein intake should be a minimum of 1.1 g/kg per day.
4. Mediterranean diet recommend to have daily 2 servings vegetables, 3 servings fruits, 3 servings skimmed dairy products and whole grain cereals. 2–3 servings/day of legumes per week, moderate to high consumption of fish and a low consumption of red and processed meats and avoiding refined grains.
5. A high dose of Vit. D (about 50,000 units every 2 weeks) is recommended.

### Nutrition for people with diabetes in critical care sitting

Hyperglycemia is common in critically ill patients and stress-induced responses to illness or injury is itself diabetogenic. Elevated blood glucose is associated with poor outcomes, including an increased infection risk [84]. In our region with a high prevalence of diabetes, managing nutrition is crucial. Reduced carbohydrate diets are recommended for type 2 diabetes [85].

When prescribing enteral nutrition for ICU patients with diabetes, consider tube feed type and rate. Standard formulas contain 50–55% calories from carbs, 30–35% from lipids, and 15–20% from protein [86]. Many of the non-diabetic formulae can be used diabetes, if blood glucose is appropriately monitored and controlled. Diabetes-specific formulas (DSFs) have relatively lower total carbs (35–40%) with more fiber and monounsaturated fats [87]. While ASPEN doesn’t directly address DSFs, ESPEN recognizes the potential benefits of using diabetes-specific formulas for ICU patients with diabetes [86, 88]. Synchronize nutrition support with insulin gives the optimal glycemic control [89].

Blenderised feeds are cost-effective in developing countries. They offer advantages over commercial formulas, containing phytochemicals and fiber. However, they can cause complications due to viscosity. Commercial enteral nutrition offers concentrated nutrients, including all essential vitamins and minerals, in standardized quantities. It provides a convenient and safe way to meet nutritional needs but lacks phytochemicals and fibers [90–92]. In a study comparing blendarized formula with commercial formula, there was no significant difference in the influence of commercial versus blendarized feeds on the patients’ weight, skinfold thickness, or blood albumin levels [90].

Some diabetic patients experiencing autonomic neuropathy may develop symptomatic gastric atony or irregular bowel motility. In such cases, post pyloric or jejunal feeding might be required [84].

Hyperglycemia is commonly seen in patients receiving PN in ICU [92, 93]. The parenteral nutrition (PN) formula can be adjusted to prevent or manage hyperglycemia. Dextrose (D-glucose) serves as the carbohydrate source in PN. It is advisable to limit the initial dextrose infusion rate to approximately 1.5–2 mg/kg of body weight per minute to prevent hyperglycemia and the development of hepatic steatosis. Once any potential adverse effects of PN are under control, the formulas can be gradually adjusted toward target levels. [94]. Avoiding the intravenous infusion of large amounts of glucose (> 5 mg/kg/min) is Recommended [95]. Another modification to the parenteral nutrition (PN) formula that can help normalize blood glucose levels involves reducing the lipid content [96]. Additionally, adding micronutrient supplements to the PN formula may enhance glycemic control. For patients with deficiencies, replacing the trace element chromium (typically in doses of 20–40 µg/day) can improve blood glucose levels and insulin resistance. Therefore, considering empirical therapy with chromium is advisable for patients at high nutritional risk who exhibit severe insulin resistance and require substantial insulin doses or continued carbohydrate underfeeding [97].

As regards the diabetic obese patient in ICU in Arab societies, one strategy is to permissively underfeed obese patients according to BMI. When BMI is in the range of 30–50 provide EN < 60-70% of target energy measured by indirect calorimetry. When indirect calorimetry is not available, using the weight-based equation providing 11–14 kcal/ kg actual body weight per day for range of BMI 30–50 (or 22–25 kcal/kg ideal body weight IBW per day if BMI above 50. Protein should be provided also according to BMI: patients with BMI 30–40 require ≥ 2.0 g/kg IBW/d and patients with BMI > 40 require ≥ 2.5 g/kg IBW/d [98].

Efforts should be made to prevent severe hyperglycemia, mild hypoglycemia, and high glycemic variability. While increasing insulin doses is a common approach to managing hyperglycemia, it’s essential to also assess the adequacy of carbohydrate administration, especially when patients require high insulin doses for more than 24 h. In rare cases, a temporary reduction in the feeding rate may be considered.

For critically ill patients with low plasma levels of 25-hydroxy-vitamin D (less than 12.5 ng/ml or 50 nmol/l), supplementation with vitamin D3 can be beneficial [95].

### **AASD position statement**

1. Adopt an optimal diet with restricted high glycemic index foods, reduced carbohydrate content, and increased dietary fiber to mitigate insulin resistance.
2. Consider oral nutritional supplements when clinically indicated, with adjustments based on blood glucose control.
3. Utilize polymeric enteral feeds with moderate carbohydrate content, administered via slow continuous infusion while monitoring and controlling blood glucose levels.
4. Integrate blenderized feeds enriched with fruits, vegetables, whole grains, and monounsaturated fats, offering advantages like phytochemicals and fibers at a lower cost.
5. Use diabetes-specific formulas (DSFs) containing monounsaturated fat and fiber for poor glycemic control.
6. When initiating PN, limit the initial dextrose infusion rate and avoid large amounts of glucose to prevent hyperglycemia.
7. When PN is indicated, daily requirements of vitamins and trace elements should be prescribed as part of PN regimen.
8. Supplement with chromium in patients at high nutritional risk with severe insulin resistance.
9. Avoid glycemic variability, targeting a blood glucose concentration of 140–180 mg/dL in critically ill adults.
10. Adjust protein requirements based on the presence of diabetic complications, such as renal impairment.
11. In obese patient with diabetes, As indirect calorimetry is not widely available in our region, so, energy intake can be based on “adjusted body weight”. If urinary nitrogen losses or lean body mass determination are not available, protein intake can be calculated as 1.3 g/kg “adjusted body weight”/d.
12. For obese patients, consider permissive underfeeding or hypo-caloric high protein feeding if hyperglycemia is poorly controlled.
13. Measure and supplement vitamin D in critically ill patients with documented deficiency.

### Diet for cancer patients with diabetes

The coexistence of cancer and diabetes is a significant challenge, with nearly 1 in 5 people with cancer also suffering from diabetes. In the Middle East and North Africa, 4.7% of cancer-related deaths in 2019 were attributable to high fasting plasma glucose [99, 100].

Cancer and its treatments can restrict food intake and cause weight loss through various mechanisms, including anxiety, anorexia, taste changes, swallowing difficulties, nausea, and gastrointestinal issues. Meanwhile, people with diabetes are at increased risk for certain cancer types due to shared pathophysiological mechanisms [101, 102].

Secondary diabetes is common in cancer patients and can result from stressful cancer treatments. In most cases, it is reversible, but 20% may progress to Type 2 diabetes. Type 1 diabetes is rare among cancer patients. Those with Type 1 or Type 2 diabetes often need to adjust their medication during cancer treatment. Additionally, Type 3c diabetes occurs in pancreatic cancer patients due to surgery affecting hormone production, managed with pancreatic enzyme replacement therapy (PERT) [103]. Widespread use of immune therapy in cancer treatment is associated with a new type of permanent insulin dependent diabetes [104].

Chronic diseases pose a significant challenge for the nutrition of cancer patients. During chemotherapy, 10-30% of patients may experience hyperglycemia, which is usually temporary but can have long-term effects [105]. Targeted therapies like Tyrosine kinase inhibitors (TKIs) can affect glucose metabolism, leading to hypo- or hyperglycemia. Everolimus, an mTOR inhibitor, is associated with hyperglycemia, varying by tumor type with the highest rate in renal cell carcinoma and the lowest in neuroendocrine tumors (NETs), breast and hepatocellular carcinomas. Recorded increase in disease free survival rate associated with somatostatin analogues (SSAs) in NETs is attributed to reduction in growth hormone and insulin-like growth factor-I (IGF-I) [106].

The simultaneous occurrence of diabetes and cancer necessitates a comprehensive approach to enhance patients’ overall well-being, treatment results, and quality of life, while minimizing diabetes-related complications and toxicities associated with cancer treatment [107]. Based on international guidelines for cancer patients care [108–112] applied globally as well as in the Arab region.

### AASD position statement are the following

1. Apply the Co-management model “Onco-Nutri MDT” to ensure that cancer patients with diabetes receive safe, timely effective and efficient care by both oncology and medical clinical nutrition complimentary perspectives.
2. Assess the nutritional status and detect disturbances at diagnosis using Nutri-screening tools with additional patient-tumor specific data age, performance status, disease stage, and treatment intentions.
3. During management, schedule regular visits to the onco-nutri clinic to monitor treatment related adverse effects, improve quality of life (QOL) or share best supportive care (BSC) to terminally ill patients.
4. Eat small frequent portions 5–6 meals per day which reduces anorexia and maintains the blood sugar within the normal range.
5. Closely monitor blood glucose levels and use intravenous insulin as the preferred treatment for hyperglycemia.
6. Administer appropriate medical treatment as needed to restore circulatory volume and the extracellular compartment. This includes lowering blood glucose levels, normalizing plasma osmolality, and correcting any electrolyte imbalances.
7. Encourage oral intake of naturally sweet foods as banana to improve appetite, as well as bitter substances like lemon juice and vinegar to stimulate saliva production. The patient may want to add some spices and herbs to food to perk up the taste while their unique flavors may substitute for salt.
8. Keep healthy snacks within reach of patients, as sliced cucumber with peanut butter or white cheese, yogurt with fruits, and whole-grain bread. With progressive mucositis, xerostomia, and dryness of mouth, preferably shift to semisolid soft processed or liquid food. Palatable oral nutrition supplements ONS are advised in anorexia and loss of appetite.
9. In weight-losing cancer patients (H&NC, CRC, cachexia or sarcopenia) with insulin resistance, we recommend increasing the ratio of energy and shift from fat to energy from carbohydrates. It is worth noting that there are currently no drugs approved by the Food and Drug Administration FDA for sarcopenia.
10. Entral nutrition EN is introduced when oral intake is temporarily insufficient or absent as during concurrent radiation and chemotherapy for H&N cancer, Rectal cancer, metastatic CRC. Using preferably nasal-gastric tube NGT, or gastrostomy tube. The Clinical Nutrition medical consultant is to assess the response on daily basis to supply the appropriate personalized nutrients hospital based the at home.
11. In situations where neither ONS nor EN are efficient or tolerable, shift to automated TPN offering substitutes admixture under complete sterile conditions to immune-compromised patients using formulas tailored according to daily needs. Central venous catheter is surgically inserted, if not accessible a peripheral venous catheter is placed in the arm usually used for short-term parenteral feeding, then taper gradually to mixed PN and ONS till patient is capable of ON.
12. A patient education and guidance to maintain a healthy lifestyle of life with adjustment of dietary habits to avoid weight gain or loss while under anti-cancer management.
-GVHD induced by BMT can affect the patient’s ability to eat and/or essential nutrients absorption.
14. For Short bowel syndrome, no role for the choice is between EN or PN at home using a notebook for daily documentation to assess for efficacy at the regular hospital visits and monitor the supply.
15. Criteria for withholding nutrition support in patients with end of life disease are: 1-short estimated life expectancy (fewer than 2–3 months), 2-poor performance status as determined by a Karnofsky score < 50% or an ECOG Status of > 2, 3-severe organ dysfunction, 4-uncontrolled symptoms, or 5-patient refusal.
16. There are very limited data testing the impact of exercising, or changes in diet composition during cancer treatment on cancer recurrence or mortality. Yet, lifestyle changes may lead to improvements in quality of life (QoL) and treatment-related long term adverse effects.
17. When patients inquire about dietary interventions like ketogenic or low-carbohydrate diets, low-fat diets, functional foods, or intermittent fasting to improve their quality of life (QoL), the response often revolves around the lack of evidence supporting their efficacy.
18. Neutropenic diets (specifically diets that exclude raw fruits and vegetables) are not recommended to prevent infection in patients with cancer during active treatment.

### Limitations of the study

We should mention some limitations of our current consensus. Diabetes with other chronic medical condition such as neurological, respiratory, autoimmune not covered, incomplete Coverage of all the previous literature regarding nutrition of diabetic persons with different medical and surgical situations, the potential for overgeneralization covering all Arab countries in their diet habits, and unlike meta-analyses, reviews do not typically involve quantitative synthesis of data, limiting the ability to draw robust conclusions.

## Conclusion

International guidelines cannot be directly applied to MENA region due to cultural and dietary differences. Recommendations should be adapted to suit local habits, preferences, and socioeconomic status and consideration of traditional foods and practices is essential. Our position statement shares valuable insights into managing diabetes in special situations and diverse clinical settings within Arab countries. It offers practical recommendations and tips for healthcare practitioners who treat people with diabetes by considering the unique context of their Countries.

## Data Availability

No datasets were generated or analysed during the current study.

## Abbreviations

GDM: Gestational diabetes
GFR: estimated glomerular filtration rate
GVH: graft vs. host
H&NC: head & neck cancer
HEI: healthy eating index
IBW: ideal body weight
ICU: intensive care unit
Kg: kilo gram
LC: low carbohydrate
LPD: low protein diet
MASH: Metabolic dysfunction-associated steatohepatitis
MASLD: metabolic dysfunction-associated steatotic liver disease
MedD: Mediterranean diet
MENA: Middle East and North Africa
MUFA: mono unsaturated fat
NAFLD: non-alcoholic fatty liver disease
NASH: non-alcoholic steatohepatitis
NGT: nasal gastrin tube
ONS: oral nutritional supplement
PN: parenteral nutrition
PUFA: poly unsaturated fat
PY: probiotic yogurt
QOL: quality of life
T1D: type1 diabetes
T2DM: type 2 diabetes mellitus
TKIs: Tyrosine kinase inhibitors
UAE: united Arabs of emirate
Vs: Versus
ω-3 FA: omega 3 fatty acid
